# Supplementary material for: Chemometric Differentiation of Sole and Plaice Fish Fillets Using Three Near-Infrared Instruments
Source: Foods. 2022 Jun 2;11(11):1643. doi: 10.3390/foods11111643 (PMC9180159; doi:10.3390/foods11111643)
Supplement: Supplementary file 1 [file foods-11-01643-s001.zip › foods-1717954-supplementary.pdf]

foods

MDPI

Supplementary Materials

Chemometric Differentiation of Sole and Plaice Fish Fillets Using Three Near-Infrared Instruments

Nicola Cavallini [1,*,†], Francesco Pennisi [2,†], Alessandro Giraudo [1], Marzia Pezzolato [2], Giovanna Esposito [2], Gentian Gavoci [1], Luca Magnani [3], Alberto Pianezzola [3], Francesco Geobaldo [1], Francesco Savorani [1,‡] and Elena Bozzetta [2,‡]

1 Department of Applied Science and Technology, Politecnico di Torino, Corso Duca degli Abruzzi 24, 10129 Turin, Italy; alessandro.giraudo@polito.it (A.G.); gentian.gavoci@polito.it (G.G.); francesco.geobaldo@polito.it (F.G.); francesco.savorani@polito.it (F.S.)
2 Istituto Zooprofilattico Sperimentale del Piemonte, Liguria e Valle d'Aosta, Via Bologna 148, 10154 Turin, Italy; francesco.pennisi@izsto.it (F.P.); marzia.pezzolato@izsto.it (M.P.); giovanna.esposito@izsto.it (G.E.); elena.bozzetta@izsto.it (E.B.)
3 Esselunga S.p.A., Via Giambologna 1, 20096 Limito di Pioltello (MI), Italy; luca.magnani@esselunga.it (L.M.); alberto.pianezzola@esselunga.it (A.P.)
† These authors contributed equally to this work.
‡ These authors contributed equally to this work.
* Correspondence: nicola.cavallini@polito.it; Tel.: +39-011-0904713

The Table of Contents:

Figure S1. Example of raw NIR spectra of cuttlefish and musky octopus samples acquired using SCiO, MicroNIR and MPA instruments.

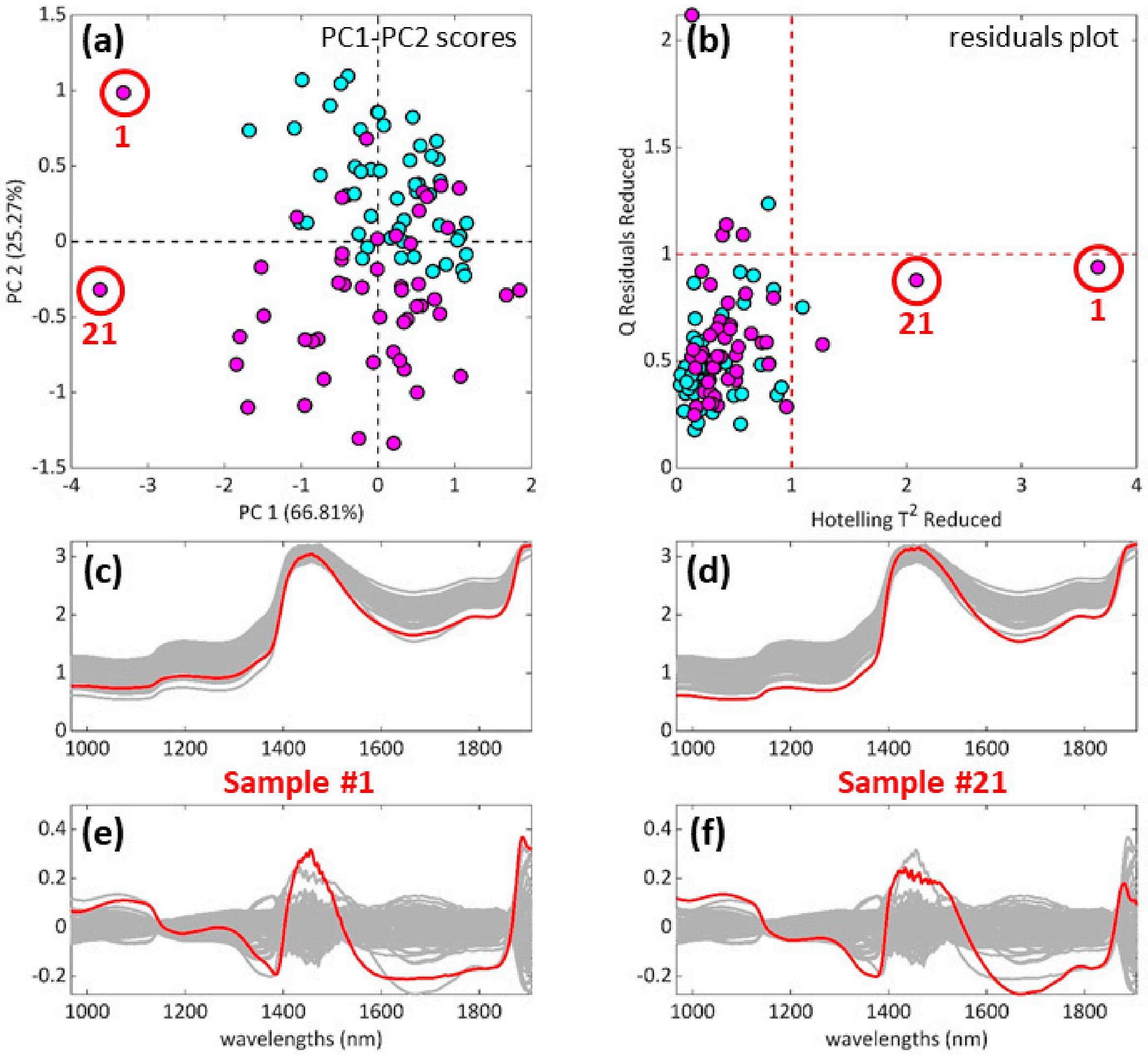

Figure S1. Samples #1 and #21 of the MPA dataset were identified as outliers and removed. They showed a strange behaviour already by inspecting the PC1-PC2 scores (a), as they were found far from the rest of the samples, and sample #1 also lied closer to the samples of the other class. By inspecting the residuals plot of the PCA model (b), it was found that the samples had rather high contributions in T^2, thus confirming the presence of "extreme" information, also highlighted by plotting their experimental profiles (c,e = raw, d,f = preprocessed with mean center).
